# The use of probiotics as a supplementary therapy in the treatment of patients with asthma: a pilot study and implications

**DOI:** 10.6061/clinics/2019/e950

**Published:** 2019-08-06

**Authors:** Jonatas Christian Vieira Moura, Isabel Cristina Gomes Moura, Guilherme Rache Gaspar, Guilherme Matos Serretti Mendes, Bernardo Almeida Vial Faria, Nulma Souto Jentzsch, Maria do Carmo Friche Passos, Amanj Kurdi, Brian Godman, Alessandra Maciel Almeida

**Affiliations:** I Programa de Pos Graduacao em Ciencias da Saude, Faculdade de Ciencias Medicas de Minas Gerais, Belo Horizonte, MG, BR; II Departamento de Pediatria, Universidade Federal de Minas Gerais, Belo Horizonte, MG, BR; III Faculdade de Medicina, Faculdade de Ciencias Medicas de Minas Gerais, Belo Horizonte, MG, BR; IV Faculdade de Medicina, Faculdade de Medicina de Barbacena, Barbacena, MG, BR; V Pharmacoepidemiology and Pharmacy Practice, Strathclyde Institute of Pharmacy and Biomedical Science, University of Strathclyde, Scotland, UK; VI Department of pharmacology, College of Pharmacy, Hawler Medical University, Erbil, Iraq; VII Division of Clinical Pharmacology, Karolinska Institute, Stockholm, Sweden; VIII Department of Public Health Pharmacy and Management, School of Pharmacy, Sefako Makgatho Health Sciences University, Garankuwa, South Africa

**Keywords:** Respiratory Hypersensitivity, Asthma, Public Health, Respiratory Function Tests, Probiotics, Brazil

## Abstract

**OBJECTIVES:**

Evaluate the use of probiotics as an additional therapy in the treatment of children and adolescents with asthma in Belo Horizonte, MG-Brazil.

**METHODS:**

A pilot longitudinal, experimental and nonrandomized study with 30 patients from six to 17 years old from Belo Horizonte. In the baseline appointment, all patients received beclomethasone, and one group also received a probiotic containing *Lactobacillus reuteri* (n=14). The patients were reassessed after at least 60 days with the Asthma Control Test, spirometry and self-report of the symptoms they experienced associated with asthma.

**RESULTS:**

A predominance of male patients (56.7%) and a mean age of 10.6 years were observed. The groups using probiotics did not differ in terms of sex, age or atopy. In the longitudinal evaluation, an increase in the Asthma Control Test scores and a reduction in the number of symptoms were observed in the probiotic group. There was an increase in the peak expiratory flow among those who used probiotics.

**CONCLUSIONS:**

This pilot study supports the hypothesis that the administration of probiotics as a supplementary therapy for the treatment of children and adolescents with asthma improves the clinical condition of the patients. Further studies are needed to confirm the efficacy of probiotics in asthma treatment.

## INTRODUCTION

Asthma involves inflammation of the airway that leads to a variable obstruction of the intrapulmonary airflow, leading to recurrent episodes of breathlessness, wheezing, chest tightness and coughing 1,2. Asthma is the most common chronic disease in childhood 3, and it is estimated that up to 14% of children and 8.6% of adults worldwide have symptoms of this pathology 4. There is a low mortality rate associated with asthma 3; however, it imposes considerable financial and social costs and is a prevalent cause of disability 5.

According to the guidelines of the Global Initiative for Asthma 2, the primary goal of asthma treatment is to obtain optimal control of the disease, with minimal or no day and night symptoms, no limitations on physical activity, a minimum need for medication for the relief of symptoms, normal or nearly normal pulmonary function, and the absence of exacerbations. These goals must be obtained with the use the lowest possible amount of medication, especially corticosteroids, according to a phased plan that takes into consideration the control of the disease, its severity and future risks 1,6. The use of probiotics arises as a possible additional therapy.

Probiotics are defined as "live microorganisms that when administered in adequate amounts, confer benefits to the host's health" 7,8. The use of these organisms could provide benefits to the patient's immune system, leading to better control of the disease, with a reduction in symptoms and an improvement in lung function in these patients. In addition, other mechanisms of action of the probiotics include the increase in the epithelial barrier, adhesion to the intestinal mucosa, inhibition of the adhesion of pathogens, exclusion of pathogenic microorganisms by competition and anti-microorganism substance production 7-9.

Several studies have investigated the possible benefits of probiotics for the prevention and/or treatment of asthma. A pilot study conducted by Stockert et al. investigated the effects of probiotics in asthmatic children 10 and found an improvement in lung function (peak of expiratory flow [PEF]) but no impact on the patients' quality of life and use of asthma medications. Furthermore, Chen et al. 11 observed improvements in symptoms, lung function and immunological parameters in children who received probiotics. Liu et al. 12 suggested that probiotics could enhance the therapeutic effect of allergen-specific immunotherapy in patients with asthma. In studies performed in rats with allergic inflammation of the airways receiving injections of *Lactobacillus reuteri*, Forsythe et al. 13 and Karimi et al. 14 observed a mitigation of inflammation and airway hyperresponsiveness in the group of animals that received the probiotic. However, there are some studies in which there was no evidence of beneficial effects of probiotics on patients with asthma or allergies 15-20. These include the study by Giovannini et al. 15 in Italy with children with allergic asthma and/or rhinitis from two to five years. The authors found that the administration of probiotics did not promote improvements among patients with asthma with regard to time free from symptoms or the number of episodes of the disease.

Rose et al. 16, examining the impact of *Lactobacillus rhamnosus* GG ATCC 53103 in children aged 6 to 24 months with wheezing for six months and a family history of atopic diseases (in first-degree relatives), also found no association between inhaled medication need and the number of days free from symptoms among children who received or did not receive the probiotics.

Given the current controversy about the use of probiotics in children with asthma, the aim of this pilot study was to evaluate the effect of including probiotics as a therapy supplementary to treatment with the inhaled corticosteroid (ICS) dipropionate beclomethasone among children and adolescents with asthma in a secondary referral unit (SRU) of Belo Horizonte, MG-Brazil. In particular, this study investigated the effect of *Lactobacillus reuteri* because there are no previous Brazilian studies investigating its possible use in this population.

## METHODS

### Study design

This was a longitudinal, quasi-experimental pilot study with a control group involving children and adolescents with asthma aged between 6 and 17 years old and treated in an SRU in Belo Horizonte between January 2015 and December 2015.

### Study participants

Children and adolescents who were newly diagnosed with mild to moderate asthma 2 and who had not been previously prescribed an ICS or short-acting beta_2_-agonist (SABA) were included. Patients with other respiratory disorders, such as bronchiolitis obliterans, interstitial pneumonia, sickle-cell disease, cystic fibrosis, and sequelae due to complicated pneumonia, tuberculosis or primary ciliary dyskinesia, were excluded. In addition, smokers and patients with cognitive impairment in the first attempt after initiation of the study were excluded.

During the baseline appointment, all children received an initial dose and a prescription for the conventional therapy, beclomethasone (Clenil^®^ 250 μg/shot - Laboratory Chiesi, Brazil), at a dose of 250 μg, twice daily (500 μg/day) and the informed consent forms were signed. Aerolin^®^ (salbutamol sulphate 120.5 mcg, norflurane S.p. Laboratory Gsk, Brazil), at a dose of 100 μg, was prescribed for use in case of exacerbations. One group received the probiotic ProVance^®^ (Laboratory Aché, Brazil) composed of 10^8^ colony-forming units (CFU) of *Lactobacillus reuteri* DS 17938 at a dosage of one capsule (10^8^ CFU)/day 8, and the other group received a placebo (no additional treatment for asthma). The allocation was performed in accordance with the final digit of the patient record, with patients with odd numbers receiving the probiotic.

### Study outcomes

To evaluate the effects of the probiotic, the patients were reassessed within a minimum period of 60 days after the initiation of treatment. The outcomes of interest were asthma control, pulmonary function and the frequency of the main asthma symptoms.

At the two appointments, patients completed the asthma control test (ACT) 21, a spirometry with bronchodilator test and an assessment of the following symptoms: coughing, wheezing, tiredness, chest pains, night-time symptoms (all symptoms cited before occurring at night), limitations on physical activities and any school absenteeism. A skin allergy test was performed only at the baseline appointment to identify atopic patients. A list of potential adverse effects of probiotics was given to the patients and their caregivers, with the instructions to monitor their occurrence during treatment.

### ACT

Nathan et al. 22 developed the ACT to monitor the responsiveness to clinical changes. It has been validated with internal consistency. The questionnaire has been validated for use in Brazil 21 and is able to discriminate controlled from uncontrolled asthma, with good reproducibility and responsiveness of the questionnaire among Brazilian patients. The ACT has five questions and assesses the frequency of shortness of breath and general asthma symptoms, the use of rescue medications, the effect of asthma on daily functioning, and an overall self-assessment of asthma control in the last four weeks. Scores range from 5 (poor control of asthma) to 25 (complete control of asthma), and high scores reflect greater degrees of asthma control. Asthma is classified as controlled if the score is greater than 19.

### Spirometry

The spirometry test was performed using the VMI ATS system (Clement Clarke, EC0120, United Kingdom) in standing patients, with the mouthpiece of a disposable spirometer positioned in the mouth and the nostrils closed with a nose clip. The test was administered by an accredited technician. The device was calibrated daily prior to testing. The patients performed a maximum inspiration followed by a maximum forced exhalation. This procedure was repeated three times before and 15-20 minutes after inhalation of a fast-acting bronchodilator spray, Aerolin^®^ (salbutamol sulfate 120.5 mcg, norflurane S.p. Laboratory Gsk, Brazil). Measurements were performed to determine the forced vital capacity (FVC), forced expiratory volume in one second (FEV_1_), PEF and Tiffeneau index (FEV_1_/FVC). The predicted values were obtained from the equations provided by Koopman et al. 23.

### Evaluation of adverse effects

The caregivers were questioned about the occurrence of the following symptoms that could indicate any adverse reactions to probiotics. These included diarrhea, vomiting, nausea, abdominal discomfort, burning sensation in the stomach and/or a bad taste in their mouth not related to the patients' food.

### Data analysis

This was a pilot study designed to be an exploratory analysis and as a proof of concept study about the inclusion of probiotics as a supplementary treatment for asthma. There was no calculation of the necessary sample size. The findings were intended be used to calculate the sample size for a larger future study.

The categorical variables are presented as frequencies, and the numerical variables are presented as the means ± standard deviation (sd), when normally distributed (assessed via the Shapiro-Wilk test), or as the medians ± interquartile ranges (IQRs), when nonnormally distributed. To evaluate the associations among the categorical variables, Fisher's exact test was used for independent samples, and the McNemar chi-square test was used for the longitudinal analysis. For the comparison of numerical variables between two groups, Student's t tests and Wilcoxon rank-sum tests were used for independent samples and paired samples, respectively. The analyses were performed with the free program R version 3.3.2. We considered significant *p*<0.05.

### Ethical approval

This study was approved by the Research Ethics Committee of the University Hospital São José/University Medical Sciences - MG under the number CAAE 36416714.4.0000.5134.

## RESULTS

In total, 37 patients met the inclusion criteria; four patients were excluded because they were present at only one appointment (one in the probiotic group), and three were excluded because they had their second appointments fewer than 60 days after the baseline appointment (one in the probiotic group). The sample was composed of 30 children and adolescents, and 14 received the probiotic. There was a predominance of males (56.7%), the mean age was 10.6±2.5 years, and the mean body mass index (BMI) was 18.8±3 kg/m^2^. The median time between the baseline and second appointments was 63±7 days; 63.3% of the patients had previously been hospitalized, 80% had previously sought emergency care, and 70% were classified as atopic subjects according to the results of the allergy tests conducted at the baseline. Among the patients using probiotics, 50% were boys, the mean age was 11±2.5 years, the mean BMI was 19.9±3.4 kg/m^2^, 42.9% had previously been hospitalized, 64.3% had previously received emergency care, and 71.4% were atopic. There were no significant differences in patient characteristics between the control and intervention groups (Table 1).

**Figure 1:**
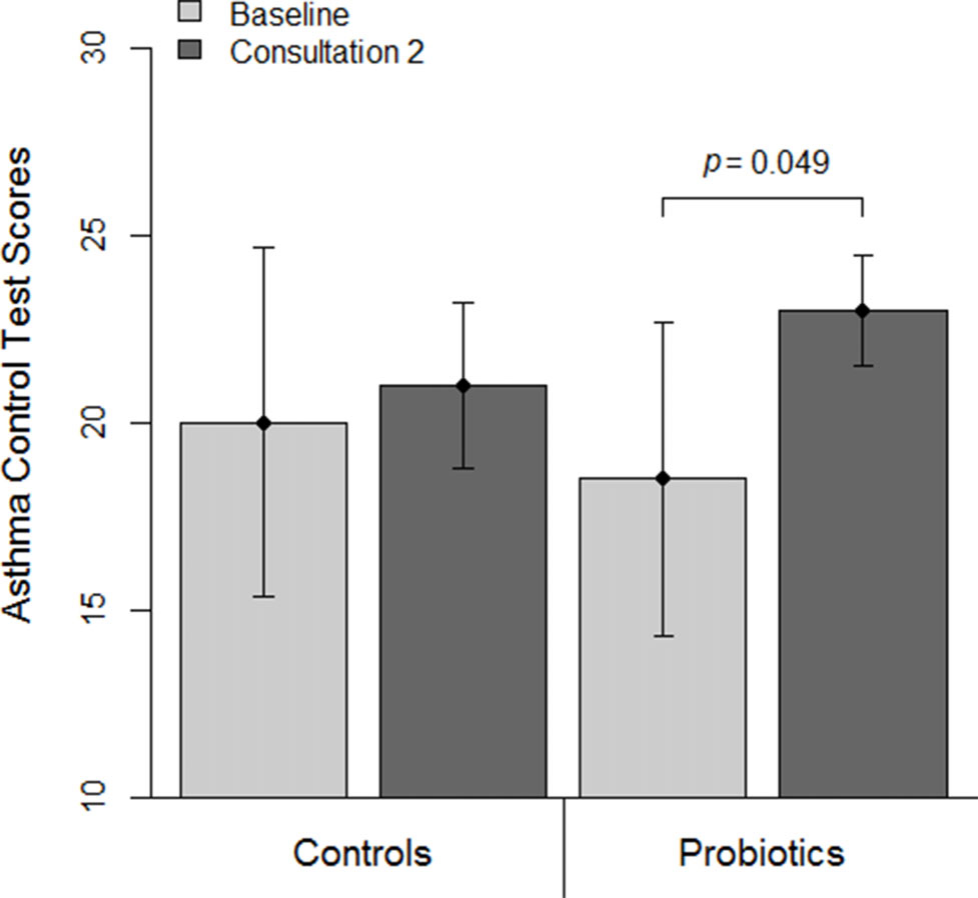
Asthma Control Test scores among patients using and not using probiotics.

In the longitudinal evaluation, there was a significant increase in ACT scores (difference mean ± sd 3.85±6.7, *p*=0.049) (Figure 1) and a significant reduction in the number of symptoms (difference mean ± sd -2.64±3.48, *p*=0.023) among the patients who used probiotics. In addition, there was a reduction in the number of patients who reported wheezing (78.6% to 21.4%, *p*=0.046). There were no significant changes in the control group (Table 2).

Regarding the parameters measured by prebronchodilator spirometry, there was an increase in PEF among patients who did not receive the probiotic (difference mean ± sd 12.47±12.33%, *p*=0.005) and those who did (difference mean ± sd 17.89±17.54, *p*=0.005) (Table 3).

The occurrences of the signs of possible adverse effects did not differ between patients who did or did not receive the probiotic.

## DISCUSSION

The groups were similar and comparable. There were no significant differences in the characteristics of the groups of patients who did or did not receive *L. reuteri.* In the group that received the supplement, an increase in ACT scores and a decrease in the number of symptoms, in particular the occurrence of wheezing, were seen, while no significant changes were observed in the control group. The spirometry results were the same in the two groups (longitudinal increase in the pre- bronchodilator PEF), which implies that probiotics had no impact on patients' spirometry tests. The possible adverse effects evaluated were not associated with the use of the supplement.

The longitudinal increase in prebronchodilator PEF indicates a possible improvement in relation to the obstruction of the lower airways by decreasing the bronchial hyperresponsiveness in all patients. These results may have been influenced by the standard treatment (beclomethasone in patients who were not previous users of either ICS or SABA), the guidelines regarding the allergic markers and climatic changes between the evaluations. The increase in PEF in the sample, for example, was expected given the application of regular ICS treatment for two months 2. Another possible explanation would be a better performance of patients on the second test due to the training they received when they performed it previously. This is notable for PEF, as it is a functional parameter that is effort dependent 24. FEV_1_ is the most important follow-up parameter in patients with asthma because its decrease increases the risk of exacerbations, regardless of the presentation of relevant symptoms 25.

The findings of this pilot study suggest that the use of probiotics may promote an attenuation of clinical symptoms because four of the five items evaluated by the ACT are related to symptoms. However, this needs to be evaluated further before any definitive statements can be made due to the small sample size. These findings are similar to those of Chen et al. 11, who showed a decrease in asthma/allergic rhinitis symptoms and improvements in FEV_1_, FVC and FEV_1_/FVC measured by spirometry and PEF measured daily among patients who received probiotics. The authors also reported a significant reduction in the levels of immunological parameters measured in peripheral blood mononuclear cells (TNF-α, IFN-γ, IL-12 and IL-13). However, we did not observe an improvement in lung function in the probiotic group in our study. This difference could be due to variations in the method used to measure lung function.

In addition, Stockert et al. 10 found a reduction in the variation in PEF in patients who used a probiotic; however, no differences were identified in the assessment of FEV_1_, quality of life and use of extra medications. The findings from Stockert et al. 10 resemble those of the present study with respect to improvements in lung function, evaluated in this case by the daily variation in PEF measured with a portable measuring instrument. The differences observed in the results between the two studies could potentially be explained by the use of different strains of probiotics, which might provide different benefits to the host 8.

Prescott and Bjorksten 26 reported that the effects of probiotics on allergic diseases can be influenced by factors such as genetic differences in microbial responses, microbial composition, individual microbiota, diet, allergic predisposition and the use of antibiotics, which could also account for the different results observed. However, in our study, we did not find an association between the probiotic effect and the allergic profile.

With regard to the limitations of this study, there were quasi-experimental characteristics (nonrandomization and lack of blinding of participants and researchers). Koopman's equations that were used to derive the predicted values of spirometry parameters were developed based on a sample that included those in the age ranges of children and adolescents. There are no national equations recently published derived from a sample of Brazilian children and adolescents up to the time of the initiation of this study. The ACT was developed for adolescents of at least 12 years old. The application of the ACT to a sample of patients younger than 12 years old in this study is mitigated because children younger than 12 years old were helped by their caregivers when completing the questionnaire.

The results of this pilot study suggest a trend towards improvement of the clinical profile of asthmatic patients who received *L. reuteri* as a supplementary therapy.

This is one of the first investigations on the efficacy of probiotics performed with Brazilian children and adolescents with asthma. However, given conflicting results in the literature, the known variability in the effects of probiotics for each strain, and the current lack of knowledge of the mechanism underlying the potential benefits of probiotics in patients with asthma, we believe that more studies are necessary, especially randomized controlled clinical trials and prospective cohort studies, to gather more evidence and knowledge of the possible potential benefits of probiotics in asthmatic patients before their routine use in children can be advocated.

## AUTHOR CONTRIBUTIONS

Moura JCV was responsible for the study design, collection of the data, interpretation of the results, manuscript writing and critical review. Moura ICG was responsible for the statistical analysis, interpretation of the results, manuscript writing and critical review. Gaspar GR, Kurdi A and Godman B were responsible for the Interpretation of the results, manuscript writing and critical review. Mendes GMS and Faria BAV were responsible for the design of the study, collection of the data, manuscript critical review. Jentzsch NS and Passos MCF were responsible for the design of the study, collection of the data, interpretation of the results, manuscript critical review. Almeida AM was responsible for the design of the study, interpretation of the results, manuscript writing and critical review.

## Figures and Tables

**Table 1:** Clinical and sociodemographic characteristics of the children and adolescents with asthma at the baseline. Belo Horizonte, 2015.

Variables	Controls (n=16)	Probiotics (n=14)	*p*-value
Male gender	10 (62.5%)	7 (50%)	0.713^F^
Follow-up time (days)	63±14	63.5±7	0.836^W^
Age (years)	10.2±2.5	11±2.5	0.383^T^
Body mass index (kg/m^2^)	17.8±2.2	19.9±3.4	0.059^T^
Prior hospitalization	13 (81.3%)	6 (42.9%)	0.057^F^
Prior emergency care	15 (93.8%)	9 (64.3%)	0.072^F^
Positive atopic allergy test	11 (68.8%)	10 (71.4%)	1.000^F^

^F^ Fisher’s exact, ^T^ Student’s t and ^W^ the Wilcoxon Mann-Whitney tests for independent samples.

**Table 2:** Symptom history of the children and adolescents with asthma stratified according to their use of probiotics. Belo Horizonte, 2015.

Variables	Measures	Controls (n=16)	Probiotics (n=14)	*p*-value
Number of symptoms	Baseline	2±5.5	4±3	0.054^W^
	2^nd^ consultation	1±2.5	1±3	0.669^W^
	*p*-value	0.251^Wp^	0.023^Wp^	
Cough	Baseline	7 (43.8%)	13 (92.9%)	0.007^F^
	2^nd^ consultation	6 (37.5%)	7 (50%)	0.713^F^
	*p*-value	1.000^Mc^	-	
Wheezing	Baseline	5 (31.2%)	11 (78.6%)	0.014^F^
	2^nd^ consultation	1 (6.2%)	3 (21.4%)	0.316^F^
	*p*-value	0.371^Mc^	0.046^Mc^	
Tiredness	Baseline	5 (31.2%)	10 (71.4%)	0.066^F^
	2^nd^ consultation	5 (31.2%)	4 (28.6%)	1.000^F^
	*p*-value	1.000^Mc^	0.077^Mc^	
Chest pain	Baseline	4 (25%)	5 (35.7%)	0.694^F^
	2^nd^ consultation	-	2 (14.3%)	0.209^F^
	*p*-value	-	0.371^Mc^	
Nighttime symptoms	Baseline	10 (62.5%)	10 (71.4%)	0.709^F^
	2^nd^ consultation	4 (25%)	5 (35.7%)	0.694^F^
	*p-*value	0.131^Mc^	0.131^Mc^	
Limitations on physical activities	Baseline	4 (25%)	5 (35.7%)	0.694^F^
	2^nd^ consultation	2 (12.5%)	4 (28.6%)	0.378^F^
	*p*-value	1.000^Mc^	1.000^Mc^	
Absent from school	Baseline	7 (43.8%)	7 (50%)	1.000^F^
	2^nd^ consultation	4 (25%)	3 (21.4%)	1.000^F^
	*p*-value	0.617^Mc^	0.221^Mc^	

^F^ Fisher’s exact, ^Mc^ the McNemar Chi-square, ^Wp^ and the Wilcoxon tests for paired samples and ^W^ the Wilcoxon Mann-Whitney test for independent samples.

**Table 3:** Pre-salbutamol spirometry parameters, as percentages of the predicted values, in the children and adolescents with asthma stratified according to their use of probiotics. Belo Horizonte, 2015.

Variables	Controls (n=16)	Probiotics (n=14)	*p*-value
FEV_1_ (L)			
Baseline	77.6±16.9	79.4±14	0.667^W^
2^nd^ consultation	81.5±8.4	85.5±8.1	0.242^W^
*p*-value	0.301^Wp^	0.147^Wp^	
FVC (L)			
Baseline	54.1±15.3	61.5±26.8	0.381^T^
2^nd^ consultation	57.4±20.5	68.1±25.7	0.254^T^
*p*-value	0.947^Tp^	0.396^Tp^	
*FEV_1_/FVC* (%)			
Baseline	104±15.1	97±21.6	0.910^W^
2^nd^ consultation	106.8±10.3	106.9±14.1	0.920^W^
*p*-value	0.476^Wp^	0.306^Wp^	
PEF (L/min)			
Baseline	75.5±19.5	74.9±17.3	0.928^T^
2^nd^ consultation	85.7±20.2	86.8±16.5	0.871^T^
*p*-value	0.005^Tp^	0.005^Tp^	

^T^ Student’s t and ^W^ the Wilcoxon Mann-Whitney tests for independent samples and ^Tp^ Student’s t and ^Wp^ the Wilcoxon tests for paired samples.

FEV_1_ = forced expiratory volume in one second; FVC = forced vital capacity, PEF = peak expiratory flow
